# Efinaconazole Topical Solution, 10%: Factors Contributing to Onychomycosis Success

**DOI:** 10.3390/jof1020107

**Published:** 2015-07-03

**Authors:** Richard A. Pollak, William J. Jo Siu, Yoshiyuki Tatsumi, Radhakrishnan Pillai

**Affiliations:** 1 San Antonio Podiatry Associates, San Antonio, TX 78229, USA; 2 Dow Pharmaceutical Sciences, a Division of Valeant Pharmaceuticals North America LLC, Petaluma, CA 94954, USA; E-Mails: wjo@dowpharmsci.com (W.J.J.S.); RPillai@dowpharmsci.com (R.P.); 3 Kaken Pharmaceutical Co. Ltd., Kyoto 607-8042, Japan; E-Mail: tatsumi_yoshiyuki@kaken.co.jp

**Keywords:** onychomycosis, efinaconazole, topical therapy, fungi, toenail

## Abstract

To provide an adequate therapeutic effect against onychomycosis, it has been suggested that topical drugs should have two properties: drug permeability through the nail plate and into the nail bed, and retention of their antifungal activity in the disease-affected areas. Only recently has the importance of other delivery routes (such as subungual) been discussed. Efinaconazole has been shown to have a more potent antifungal activity *in vitro* than the most commonly used onychomycosis treatments. The low keratin affinity of efinaconazole contributes to its effective delivery through the nail plate and retention of its antifungal activity. Its unique low surface tension formulation provides good wetting properties affording drug delivery both through and under the nail. High antifungal drug concentrations have been demonstrated in the nail of onychomycosis patients, and effectiveness of efinaconazole topical solution, 10% confirmed in two large well-controlled multicenter Phase 3 clinical studies in patients with mild-to-moderate disease.

## 1. Introduction

Onychomycosis is a progressive, common fungal infection of the nail bed, matrix or nail plate [1,2]. It is difficult to treat successfully and can be extremely recalcitrant. The disease may be managed with oral or topical medication, with topical agents having low risk of hepatotoxic side effects and the drug-drug interactions usually associated with some systemic antifungals, such as itraconazole.

Traditional formulation approaches to topical onychomycosis therapy have employed lacquers (*i.e.*, ciclopirox and amorolfine). These are organic solutions of film forming polymers. Upon application to the surface of the nail, the solvent evaporates, leaving a water resistant polymer film on the nail plate. This occlusive film acts as a reservoir from which drug is released, penetrating into and through the nail. One of the disadvantages of lacquers is that the film must then be removed, either mechanically or with organic solvents, and fresh lacquer applied to replenish this drug depot [3]; a process that can render the nail more prone to infection [4]. Indeed, ciclopirox lacquer required weekly removal of the product and it was also strongly recommended that patients see a podiatrist or their physician every few months for mechanical debridement of their toenails. However, the main concern with lacquer formulations is that efficacy in treating onychomycosis has been disappointing [5]. One reason for their low cure rates is thought to be the inability of drug to efficiently penetrate the nail plate [6,7,8].

The intrinsic properties of a drug molecule and the vehicle in which the drug is formulated are both felt to be important contributors to topical treatment success in onychomycosis. Not only must an adequate amount of drug be delivered to the infection, its antifungal activity must be maintained within the keratin-rich environment of the nail. Both penetration through the nail and resultant drug activity can be restricted or decreased through keratin binding [9,10,11].

The onychomycotic nail is visually very different to the healthy nail. Although these nails tend to be thicker [12,13], nail density and tensile strength are reduced, suggesting a more porous structure and erosion of the intracellular matrix, rendering the tissue more permeable to topically applied agents formulated in an aqueous vehicle solution [14]. Newer topical agents for onychomycosis (*i.e.*, efinaconazole and tavaborole) have been formulated as solutions. Complete cure rates of efinaconazole of 15.2% and 17.8% have been reported from two large Phase 3 clinical trials in mild to moderate onychomycosis [15].

While much has been written about the topical treatment of onychomycosis and the challenge of effective nail penetration following product application to the nail surface, the potential of other delivery routes, such as subungual application at the hyponychium, has been largely overlooked. Efinaconazole topical solution, 10% is a uniquely formulated antifungal specifically developed for the treatment of toenail onychomycosis. Its formulation has both a low surface tension and good wetting properties [16], affording the potential of both transungual and subungual delivery of active drug to the infection.

Our review highlights the *in vitro* and *in vivo* studies with efinaconazole, and presents data on transungual and subungual delivery to the site of infection in the nail bed, matrix, and nail plate in onychomycosis patients.

## 2. *In Vitro* Antifungal Activity of Efinaconazole

Most onychomycosis infections are due to dermatophyte fungi (mainly *Trichophyton rubrum* and *Trichophyton mentagrophytes* in about 80%–90% of cases) [17,18,19,20]; but can also be caused by nondermatophytes (mainly *Scopulariopsis brevicaulis*, although this can vary by country), and yeasts (mainly *Candida albicans*) [21]. Nondermatophyte infections are becoming increasingly prevalent in onychomycosis, either due to an artifact of improved diagnostic techniques or through increased awareness [22,23]. In addition, mixed infections have been reported, although their significance is less clear [24]. Therefore, an antifungal with a broad spectrum of activity is felt to be increasingly important in successfully treating onychomycosis [25].

Efinaconazole has a broad antifungal activity against dermatophytes, nondermatophytes, and yeasts. Efinaconazole demonstrated antifungal activity against *T. rubrum* and *T. mentagrophytes* (MIC_90_: 0.008–0.015 μg/mL) and *C. albicans* (MIC_50_: 0.004 μg/mL) and was more potent than the most commonly used antifungals in onychomycosis [26]. Against *T. rubrum* and *T. mentagrophytes*, efinaconazole had comparable activity (1 to 4-fold) to both amorolfine and terbinafine, and higher activity (8 to 64-fold) than ciclopirox and itraconazole [26]. Efinaconazole was significantly more potent (*p* < 0.001) in inhibiting *C. albicans* than terbinafine, ciclopirox, itraconazole and amorolfine [26].

## 3. Keratin Affinity and Transungual Penetration *in Vitro*

The upper (dorsal) layer of the nail is only a few cell layers thick but consists of hard keratin, and is the main barrier for drug permeation into and through the nail plate [27]. Many antifungal drugs are known to possess high keratin affinity, a property that may have a deleterious effect on their nail penetration and efficacy [28]. Keratin-bound drug does not contribute to the concentration gradient that would otherwise increase drug penetration, resulting in accumulation on the surface layers of the nail, decreased penetration into the deeper layers and the nail bed, and reduced antifungal activity [29]. Terbinafine, for example, is 98.9% keratin-bound *in vitro* [28] and clinical trials through Novartis with a topical formulation have demonstrated poor efficacy results in onychomycosis despite its high *in vitro* antifungal activity [30]. When ciclopirox was applied to the nail for 14 days, penetration into the ventral side of the nail was 2 to 4 orders of magnitude lower than on the dorsal side [31].

Efinaconazole has relatively lower binding to keratin, and faster release of bound drug from keratin when compared to ciclopirox and amorolfine. The efinaconazole free-drug concentration in keratin suspensions was 14.3% ± 0.4%, significantly higher than those seen with ciclopirox or amorolfine (0.7% ± 0.0%, and 1.9% ± 0.2%, respectively, *p* < 0.001) [28].

The low keratin affinity of efinaconazole contributes to its favorable nail penetration [28]. *In vitro* nail permeation of efinaconazole was much greater than that seen with ciclopirox following a single application to human nails, whereas amorolfine levels were not detectable [28]. The cumulative permeated amounts of efinaconazole were 2.94 ± 3.91 μg/cm^2^ and 6.53 ± 8.15 μg/cm^2^ (mean ± SD), respectively at day 7 and 14; compared with 0.326 ± 0.590 μg/cm^2^ and 4.57 ± 6.89 μg/cm^2^ (mean ± SD), respectively for ciclopirox [28].

## 4. Transungual Penetration in Onychomycosis Patients

Onychomycosis patients were treated with efinaconazole topical solution, 10% for 28 days, with a follow-up at two weeks after the last drug application. Drug concentrations in the toenail (5.9 ± 5.1, 6.0 ± 3.9, and 3.1 ± 3.2 mg/g at weeks 2, 4 and 6, respectively) were four orders of magnitude higher than MIC values of efinaconazole against *T. rubrum* and *T. mentagrophytes*. Concentrations of efinaconazole in the nail were not influenced by the presence of disease or nail thickness, and were maintained at high levels post-treatment (week 6) suggesting that the nail plate and nail bed continue to be exposed to inhibitory drug concentration for a period of time after treatment has ceased [32].

## 5. Subungual Penetration in Onychomycosis Patients

One possible explanation of the favorable clinical efficacy seen with efinaconazole topical solution, 10% is that it may not rely solely on transungual delivery to the site of infection. The unique formulation provides low surface tension and good wetting properties [16]. As a result, as well as penetrating through the nail plate, the formulation spreads along the sides of the nail, under the cuticle, and through the hyponychium, enhancing drug delivery into the nail unit.

**Figure 1:**
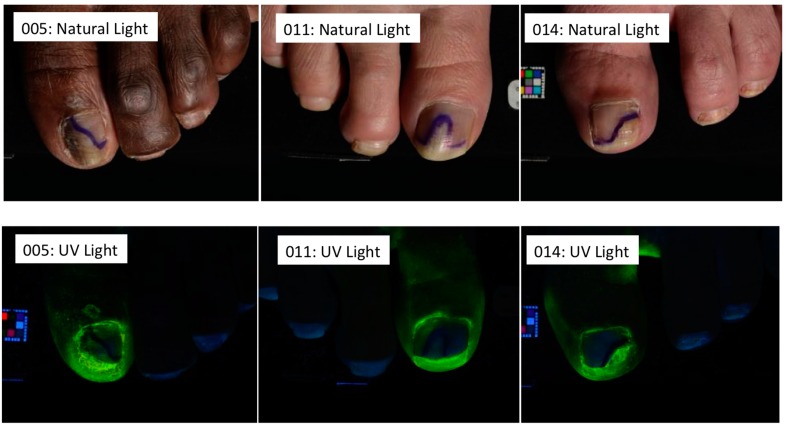
Baseline (natural light) and post clipping (UV light) photographs of three representative patients. Subject 005: 55% nail involvement; subject 011: 35% nail involvement; subject 014: 40% nail involvement. In all subjects efinaconazole vehicle solution was applied to the hyponychium only. See [33] for detailed methodology and results.

In a study of 11 onychomycosis patients, vehicle solution was applied solely to the hyponychium and was shown to spread into the subungual space between the nail plate and nail bed, reaching the site of infection [33] (Figure 1).

## 6. *In Vitro* Antifungal Activity under the Nail Plate

The antifungal activity of efinaconazole solution (and ciclopirox and amorolfine nail lacquers) against *T. rubrum* in the underlying nail plate of isolated healthy human nails was evaluated *in vitro* to determine whether the drug penetrates across the nail plate and exerts an antifungal effect on dermatophytes under the nail plate [28]. Single application of efinaconazole to the surface of toenails inhibited the growth of *T. rubrum*. The lack of growth inhibition with the comparator lacquers may be due to the poor nail penetration of amorolfine (despite its high antifungal activity), and low antifungal activity of ciclopirox nail lacquer (despite adequate nail penetration) [28].

## 7. *In Vitro* Antifungal Activity in the Presence of Keratin

The *in vitro* onychomycosis model above (Section 6) evaluated the growth inhibitory effect of drug under the nail but not necessarily fungicidal activity. Thus, viable cell counts were further determined after incubation in keratin media, which has keratin as the sole energy nutrient and mimics the environment of the nail bed. The fungicidal activity of efinaconazole against *T. mentagrophytes* in keratin media was compared with ciclopirox and amorolfine to determine fungicidal potency inside the nail and in the nail bed. Efinaconazole showed fungicidal activity at a lower concentration than amorolfine and ciclopirox [28]. In addition, unlike amorolfine and terbinafine, which have higher keratin affinity, the growth inhibitory activity of efinaconazole was not affected by keratin [10]. These higher antifungal activities of efinaconazole in the presence of keratin may be reflective of its higher free (unbound) concentration relative to the comparator drugs. The relatively high antifungal activity of efinaconazole in the presence of keratin would lead to antifungal activity in the affected nail areas.

## 8. *In Vivo* Assessment of Efficacy in Guinea Pig Model of Onychomycosis

The comparative efficacy of efinaconazole topical solution, 10% was investigated *in vivo* in a refractory guinea pig onychomycosis model where nails were infected with *T. mentagrophytes*, and infection established over 4 weeks prior to treatment. The viable cell counts in nails treated with efinaconazole topical solution, 10% were significantly lower than those treated with ciclopirox and amorolfine nail lacquers (*p* < 0.01 and *p* < 0.001, respectively) following repeated treatment over four weeks [28].

## 9. Conclusions

**Figure 2:**
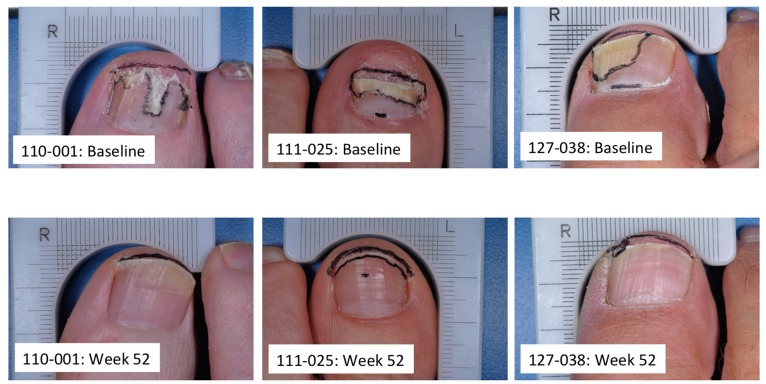
Baseline and study end (week 52) photographs of three representative patients. Subject 110–001: 25% nail involvement at baseline and 0% nail involvement at week 52; subject 111–025: 50% nail involvement at baseline and 10% nail involvement at week 52; subject 127–038: 45% nail involvement at baseline and 1% nail involvement at week 52. All subjects treated with efinaconazole topical solution, 10% daily for 48 weeks. See [15] for detailed methodology and results.

Onychomycosis is a very common fungal infection that can be difficult to treat successfully. To provide an adequate therapeutic effect, a topical antifungal must be able to reach the site of infection in sufficient concentration to eradicate the infecting pathogens. Efinaconazole is a broad spectrum antifungal with a higher *in vitro* potency than other agents frequently used to treat onychomycosis. Its low keratin affinity and unique formulation affords both transungual and subungual delivery to the site of infection in the nail bed, matrix and nail plate. Effective antifungal activity has been demonstrated experimentally *in vitro* and *in vivo* onychomycosis models, and high drug nail concentrations measured in onychomycosis patients that are well above antifungal concentrations. Clinical effectiveness of efinaconazole topical solution, 10% has been confirmed through two adequate and well-controlled large multicenter studies in 1655 patients with mild to moderate onychomycosis [15] (Figure 2). The excellent therapeutic effects of efinaconazole may be attributable to the high nail penetration, potent antifungal activity under the nail plate, strong fungicidal activity in the presence of keratin, and a multi-directional delivery approach to treatment.
